# Develop and Apply Electrocardiography-Based Risk Score to Identify Community-Based Elderly Individuals at High-Risk of Mortality

**DOI:** 10.3389/fcvm.2021.738061

**Published:** 2021-10-08

**Authors:** Tzu-Pin Lu, Amrita Chattopadhyay, Kuan-Chen Lu, Jing-Yuan Chuang, Shih-Fan Sherri Yeh, I-Shou Chang, Ching-Yu Julius Chen, I-Chien Wu, Chih-Cheng Hsu, Tzu-Yu Chen, Wei-Ting Tseng, Chao Agnes Hsiung, Jyh-Ming Jimmy Juang

**Affiliations:** ^1^Department of Public Health, Institute of Epidemiology and Preventive Medicine, National Taiwan University, Taipei, Taiwan; ^2^Bioinformatics and Biostatistics Core, Center of Genomic and Precision Medicine, National Taiwan University, Taipei, Taiwan; ^3^Department of Medical Laboratory Science and Biotechnology, China Medical University, Taichung, Taiwan; ^4^Department of Environmental and Occupational Medicine, National Taiwan University Hospital Hsin-Chu Branch, Hsinchu, Taiwan; ^5^Institute of Population Health Sciences, National Health Research Institutes, Zhunan, Taiwan; ^6^Cardiovascular Center and Division of Cardiology, Department of Internal Medicine, National Taiwan University Hospital and National Taiwan University College of Medicine, Taipei, Taiwan

**Keywords:** prevalence, long-term prognosis, electrocardiographic abnormality score, Han Chinese population, community-based

## Abstract

With an aging world population, risk stratification of community-based, elderly population is required for primary prevention. This study proposes a combined score developed using electrocardiographic (ECG) parameters and determines its long-term prognostic value for predicting risk of cardiovascular mortality. A cohort-study, conducted from December 2008 to April 2019, enrolled 5,380 subjects in Taiwan, who were examined, using three-serial-12-lead ECGs, and their health/demographic information were recorded. To understand the predictive effects of ECG parameters on overall-survival, Cox hazard regression analysis were performed. The mean age at enrollment was 69.04 ± 8.14 years, and 47.4% were males. ECG abnormalities, LVH [hazard ratio (HR) = 1.39, 95% confidence intervals (CI) = (1.16–1.67), *P* = 0.0003], QTc [HR = 1.31, CI = (1.07–1.61), *P* = 0.007] and PR interval [HR = 1.40, CI = (1.01–1.95), *P* = 0.04], were significantly associated with primary outcome all-cause death. Furthermore, LVH [HR = 2.37, CI = (1.48–3.79), *P* = 0.0003] was significantly associated with cardiovascular death, while PR interval [HR = 2.63, CI = (1.24– 5.57), *P* = 0.01] with unexplained death. ECG abnormality (EA) score was defined based on the number of abnormal ECG parameters for each patient, which was used to divide all patients into sub-groups. Competing risk survival analysis using EA score were performed by using the Gray's test, which reported that high-risk EA groups showed significantly higher cumulative incidence for all three outcomes. Prognostic models using the EA score as predictor were developed and a 10-fold cross validation design was adopted to conduct calibration and discrimination analysis, to establish the efficacy of the proposed models. Overall, ECG model could successfully predict people, susceptible to all three death outcomes (*P* < 0.05), with high efficacy. Statistically significant (*P* < 0.001) improvement of the c-indices further demonstrated the robustness of the prediction model with ECG parameters, as opposed to a traditional model with no EA predictor. The EA score is highly associated with increased risk of mortality in elderly population and may be successfully used in clinical practice.

## Introduction

The world's population is gradually aging as every country in the world is facing an increase in the number and proportion of older people in its population. Based on data from the United Nations' World Population Prospects, 1 in 11 people (9%) was over the age of 65 years in 2019, and this proportion will increase to nearly 1 in 6 people (17%) by 2050. Therefore, it is to be expected that the prevalence of diseases associated with old age, such as cardiovascular disease, will also rise. Thus, for the patients, health providers, and families, risk stratification for elderly people who have or are susceptible to chronic diseases such as hypertension, diabetes mellitus or chronic kidney diseases, is important, for primary prevention. Therefore, finding an effective, low-cost, non-invasive and readily available risk stratification tool for clinical practice is an important issue that needs to be addressed.

Although traditional cardiovascular risk factors (e.g., hypertension, diabetes or hyperlipidemia) have been identified and are widely used in clinical practice for risk stratification of cardiovascular disease development and cardiovascular events (1–3), the predictive value is less accurate in older adults than in middle-aged adults (4). An increased risk of sudden cardiac deaths has also been reported to be associated with several electrocardiographic (ECG) parameters (5–7), however, most of these parameters are used as a single predictor (e.g., QT interval) to predict clinical outcomes, resulting in limited predictive value. Combining several ECG parameters into integrated traditional risk models may improve risk stratification of cardiovascular events or mortality. This approach has not been investigated, until now, in elderly people.

Mostly, prior studies have been conducted in relatively young or middle-aged general populations (<65 years old) (5, 6, 8, 9) or in disease cohorts (10, 11) and the use of ECG parameters as predictive values of cardiovascular events for the general elderly population is scarce. The Healthy Aging Longitudinal Study (HALST) is a large, multi-site cohort study of community-dwelling older-middle-aged and elderly adults (>55 years old), with at least a 10-year follow-up, from the Han Chinese general population in Taiwan. Here, we sought to develop a combined ECG score using several ECG parameters and determine the score's long-term prognostic value for all-cause mortality, cardiovascular mortality and unexplained mortality in the HALST cohort.

## Methods

### Study Subjects: Taiwan Geriatric Health Survey population

We performed a prospective community-based cohort study in Taiwan, the HALST study (Supplementary Material), a community-based cohort study, that enrolled 5,380 study participants (12–14). Participants were enrolled from December 2008 through March 2013, and were followed up until April 2019. We collected three serial 12-lead ECGs and other relevant clinical and demographic information prospectively at the time of enrollment according to the study protocol. All analysis were conducted between May 2019 and September 2020.

We excluded individuals with a history of any cancer, significant heart diseases (e.g., documented coronary artery disease, severe heart failure (New York Heart Association class III/IV), myocardial infarction, severe valvular diseases, any kinds of cardiomyopathies and infectious cardiac diseases), ventricular conduction delay (2nd or 3rd degree atrioventricular block), atrial fibrillation or flutter, congenital short or long QT syndrome (15, 16), Brugada-type ECG (17), catecholaminergic polymorphic ventricular tachycardia (18), ventricular pre-excitation, and implanted pacemakers. The final study cohort population consisted of 4,615 individuals, constituting a random sample representative of the entire source population. All those who died of cardiac or other causes (*n* = 87 and *n* = 683, respectively) during the follow-up (mean follow-up time 95.1 ± 21.9 months) were identified.

### Long-Term Prognosis and Follow-Up

The clinical outcomes that are considered in this study are all-cause death, cardiovascular death, and unexplained death. Death from cardiovascular causes was defined by the ICD codes I01-I02.0, I05-I09, I20-I25, I27, and I30-I52. The definition of unexplained deaths are sudden deaths with unexplained causes, occurring in an individual older than 1 year, according to 2020 AHPRS/HRS expert consensus statement (19). Taiwan maintains extensive administrative registers that record every death in the country. Two clinicians blinded to the ECG results determined the causes of death by examining death certificates from the National Taiwan Institute of Health and Welfare. The causes of death were adjudicated by a committee of experienced cardiologists, who were blinded to the data from the electrocardiographic analyses.

### ECG Measurement and Definition of Abnormality Score

The 12-lead ECGs (Hewlett Packard, USA) were recorded at the standard settings of 10 mm/mV and 25 mm/s. PR and QRS intervals were computed automatically, while QTc was computed using Bazett's formula. We selected ECG variables, that are associated with mortality and relatively easily obtained in clinical practice, for risk analysis. The criteria for abnormality for each of the ECG parameters were defined as follows: PR interval >220 ms, QRS duration >110 ms, spatial QRS-T angle >90°, corrected QT interval (QTc) >450 ms in men or >460 ms in women, heart rate > 80/min, ST segment elevation in lead aVR, early repolarization pattern (ERP) in inferior or lateral leads (lead II, III, aVf; V4–6), fragmented QRS (fQRS) (20, 21) and left ventricular hypertrophy (LVH) by Sokolow-Lyon criteria or modified Cornell Criteria (22, 23). Each abnormal ECG variable counted for 1 point.

ECGs were displayed on a 24-in computer screen in multiple formats, enabling careful classification of slurring on the downslope of the R and J waves. All ECGs were analyzed and interpreted independently in random order by two trained cardiologists, who were blinded to clinical data and follow-up status. In case of divergent results, a third blinded cardiologist re-interpreted the ECG, and a preliminary decision on each ECG parameters was achieved by majority vote. After the preliminary decision on each ECG parameters, two trained cardiologists jointly re-assessed, and a final decision was reached by consensus. The inter-observer agreement was *k* = 0.97, and agreement proportion = 0.98.

### Statistical Analysis

#### Cumulative ECG Abnormality Score

All-cause death, cardiovascular (CV) death, and unexplained death were considered as the study endpoints (Supplementary Materials). All-cause death, is considered as the primary study endpoint as it is comprised of CV death, unexplained death and deaths due to other causes. A Cox proportional hazards model was used to estimate the hazard ratio and 95% confidence intervals (CI) of the nine ECG risk parameters (Table 1) (24) using all study samples. A univariate analysis, age- and sex-adjusted, were conducted with each ECG parameter as predictor for the three clinical outcomes, followed by a joint analysis of all ECG parameters, with an age-adjusted and sex-adjusted model. As each of the ECG parameters would generally have a low predictive power, explaining only a small percentage of the variance, we proceeded to include all nine parameters to calculate a primary, cumulative ECG based score, named as ECG abnormality (EA) score to increase the explanatory power by looking at the joint effect of multiple ECG parameters. Subjects were stratified into four groups: (a) subjects in Group 0 showed no abnormal ECG readings, (b) Group 1's subjects exhibited one ECG abnormality, (c) Group 2's exhibited two ECG abnormalities, and (d) subjects in Group 3 showed three or more abnormalities. Additionally, a secondary EA score was developed, as a comparison, by stratifying the subjects into (i) a low-EA group (ECG abnormalities ≤1) and (ii) a high-EA group (ECG abnormalities ≥2).

**Table 1:** Association between ECG parameters and all-cause death. ^#^(*N* = 4,530).

		**Univariate model**		**Multivariate model**	
**ECG Parameter**	***N (%)***	**HR (95%CI)**	***P*** **-value**	**HR(95%CI)**	***P*** **-value**
Heart rate >80/min	452 (9.97)	1.14 (0.91−1.45)	0.27	1.07 (0.84−1.36)	0.56
Early repolarization pattern (ERP)	880 (19.42)	1.03 (0.84−1.26)	0.79	1.01 (0.82−1.24)	0.94
Fragmented QRS complex (fQRS)	1,869 (41.26)	1.01 (0.86−1.18)	0.91	1.01 (0.86−1.18)	0.97
ST elevation in lead aVR	0 (0)	NA	NA	NA	NA
Left ventricular hypertrophy (LVH)^*^	735 (16.22)	1.39 (1.17−1.67)	0.0002	1.39 (1.16−1.67)	0.0003
QRS duration >110 ms	311 (6.86)	0.97 (0.74−1.28)	0.85	0.89 (0.67−1.17)	0.42
Corrected QT interval (QTc) (ms)^*^	1,001 (22.09)	1.32 (1.09−1.59)	0.004	1.31 (1.07−1.61)	0.007
PR interval (ms)^*^	115 (2.54)	1.43 (1.03−1.98)	0.03	1.40 (1.01−1.95)	0.04
QRS-T angle >90°	88 (1.94)	1.10 (0.62−1.96)	0.73	1.12 (0.63−1.99)	0.7

# *P-value (age-adjusted and sex-adjusted) is calculated from a Cox hazard regression model. HR: hazard ratio; CI: confidence interval*;

* *P < 0.05*.

#### Competing Risk Analysis

This study consists of three events where the occurrence of one may preclude or significantly alter the probability of the other two events. Therefore, competing risk analysis, using the complete data (*N* = 4,530), were performed for each of the three events, all-cause death, CV death and unexplained death, to estimate the hazard of failing from each of the given causes (cause-specific hazard function) in the presence of other two competing risk events, respectively (25). The cumulated incidence function (CIF) (probability of failing from a specific cause *C* before time) was employed using the “survminer” (26) and “cmprsk” (27) packages in R and CIF plots were used to compare the cumulative incidence of subjects with EA scores. Gray's test was performed to elucidate whether significant differences exist in the different groups: (A) all-cause death, (B) CV death, and (C) unexplained death.

#### Development of Prognostic Models With ECG Risk Scores

The EA scores were used to propose prognostic models using all patients (*N* = 4,530) in this study, including (i) an age and sex adjusted EA score prognostic model and (ii) a multivariate-adjusted EA score prognostic model, for all three endpoints in this study. The variables included in the multivariate adjustment consisted of traditional variables from the baseline model, such as age, sex, systolic blood pressure (SBP), diastolic blood pressure (DBP), body mass index (BMI), current smoking status, hypertension, diabetes mellitus, hyperlipidemia, stroke, and chronic kidney disease. The Cox proportional hazards model was employed using the “Survival” package in R (24) to estimate the hazard ratio and the corresponding 95% CIs. Statistical tests and graphical diagnostics based on the scaled Schoenfeld residuals testing, were used to check the proportional hazards (PH) assumption for all outcomes and for all models (age and sex adjusted ECG model and Multivariate adjusted ECG model) that have been proposed in this study (28).

#### Model Evaluation Using Internal Validation Cohort

To evaluate whether EA scores can help to identify elderly people's susceptibility to death outcomes, a 10-fold cross-validation (10CV) was performed to conduct model calibration and discrimination as indicators of performance (29). The study subjects from the HALST cohort were randomly split into training and testing sets in the ratio of 9:1, and repeated 10 times, to ensure all of the 10 data folds were used as the testing data. The training data were used on the (i) a multivariate-adjusted EA score prognostic model (ECG model), and (ii) a baseline model consisting of only traditional variables adjusted by age and sex (traditional model), to estimate the coefficients of the predictor variables, and the testing data were used to validate the fitted models, for all three outcomes.

Harrell's c-index from the “Survival” package in R (24) was utilized for the discrimination analysis to evaluate the concordance of predicted and observed survival. For a prognostic model to have a robust prediction performance for a pair of patients, the patient with shorter time to event (T) should have higher risk scores (R), as assigned by the model. In this study c-indices from all 10 cross-validation runs were averaged to evaluate the models' performances for all three outcomes. Furthermore, the c-indices of the two prediction models, with and without adding ECG parameters, were compared using a bootstrapping strategy (30) to check if adding the EA score into the prediction model leads to significant improvement in the discrimination ability, as opposed to only traditional variables.

For the calibration analysis, difference between the observed and predicted mortality for both the ECG model and the traditional model, were computed as percentages, over all study subjects, by a given follow up time. Furthermore, a proportion test was conducted to check if the differences attained statistical significance (*P* < 0.01).

## Results

### Baseline Characteristics

From a total of 5,380 relatively healthy and ambulatory individuals, enrolled in this study (Supplementary Figure 1), 755 individuals were excluded due to cancer, underlying severe cardiovascular diseases (e.g., myocardial infarction or pacemaker implantation), or missing follow-up information. Finally, 4,530 individuals with complete data were used for analysis (Supplementary Table 1).

### Prognostic Value of Individual ECG Parameters

To evaluate whether ECG parameters were associated with the death outcomes, we performed a log-rank test using both univariate and multivariate approaches. As shown in Table 1, LVH, QTc and the PR interval were significantly associated with the primary outcome, all-cause death (*P* < 0.05). The hazard ratios (HRs) of LVH, QTc and the PR interval were 1.39 [95% CI = (1.16–1.67)], 1.31 [95% CI = (1.07–1.61)] and 1.40 [95% CI = (1.01–1.95)], respectively, in the multivariate model. Similarly, the LVH was a significant predictor of CV death [HR = 2.37, 95% CI = (1.48–3.79), *P* < 0.05] (Supplementary Table 2), and PR interval was the risk predictor of unexplained death [HR = 2.63, CI= (1.24– 5.57), *P* < 0.05] (Supplementary Table 3).

### ECG Abnormality Risk Groups' Association With All-Cause Mortality, Cardiovascular Mortality and Unexplained Mortality

To further evaluate whether ECG parameters can be used to stratify people into different risk groups, we divided subjects into subgroups based on how many ECG abnormal parameters they displayed. The baseline characteristics of each ECG group are summarized in Table 2. Comparison among all groups were conducted to report *p*-values (Table 2 and Supplementary Table 4).Some clinical factors showed significant differences among the 4 groups, including age, systolic and diastolic blood pressures, smoking and hypertension (*P* < 0.05).

**Table 2:** Demographic characteristics of study subjects (*N* = 4,530) divided into subgroups based on number of ECG abnormal parameters (0, 1, 2, ≥3).

**ECG Parameters**	**0**	**1**	**2**	**> = 3**	***P*** **-value**
	***N =*** **1,151**	***N =*** **1,855**	***N =*** **1,060**	***N =*** **464**	
Gender					0.09603
Male (%)	609 (52.91)	944 (50.89)	567 (53.49)	265 (57.11)	
Female (%)	542 (47.09)	911 (49.11)	493 (46.51)	199 (42.89)	
Age (years)	68.31 ± 8.06	68.73 ± 7.88	69.81 ± 8.287	70.32 ± 8.74	3.46E−07
Systolic blood pressure (mmHg)	126.28 ± 17.76	126.33 ± 17.84	131.39 ± 19.88	134.63 ± 19.44	<2e−16
Diastolic blood pressure (mmHg)	69.87 ± 10.22	69.59 ± 10.31	71.34 ± 10.89	72.91 ± 11.79	2.30e−10
Body mass index	24.39 ± 3.295	24.48 ± 3.47	24.51 ± 3.49	24.58 ± 3.75	0.735
Current smoker (%)	123 (10.68)	275 (14.82)	134 (12.64)	59 (12.71)	0.02678
Hypertension (%)	445 (38.67)	731 (39.40)	527 (49.72)	269 (57.97)	<2.2e−16
Diabetes mellitus (%)	185 (16.07)	330 (17.79)	193 (18.20)	96 (20.69)	0.1633
Stroke (%)	47 (4.08)	85 (4.58)	60 (5.66)	28 (6.03)	0.1979
Hyperlipidemia (%)	353 (30.67)	590 (31.81)	354 (33.40)	145 (31.25)	0.5754
Chronic respiratory disease (%)	26 (2.26)	59 (3.18)	35 (3.30)	16 (3.45)	0.387
Chronic kidney disease (%)	170 (14.77)	285 (15.36)	140 (13.21)	63 (13.58)	0.4034

**Figure 1:**
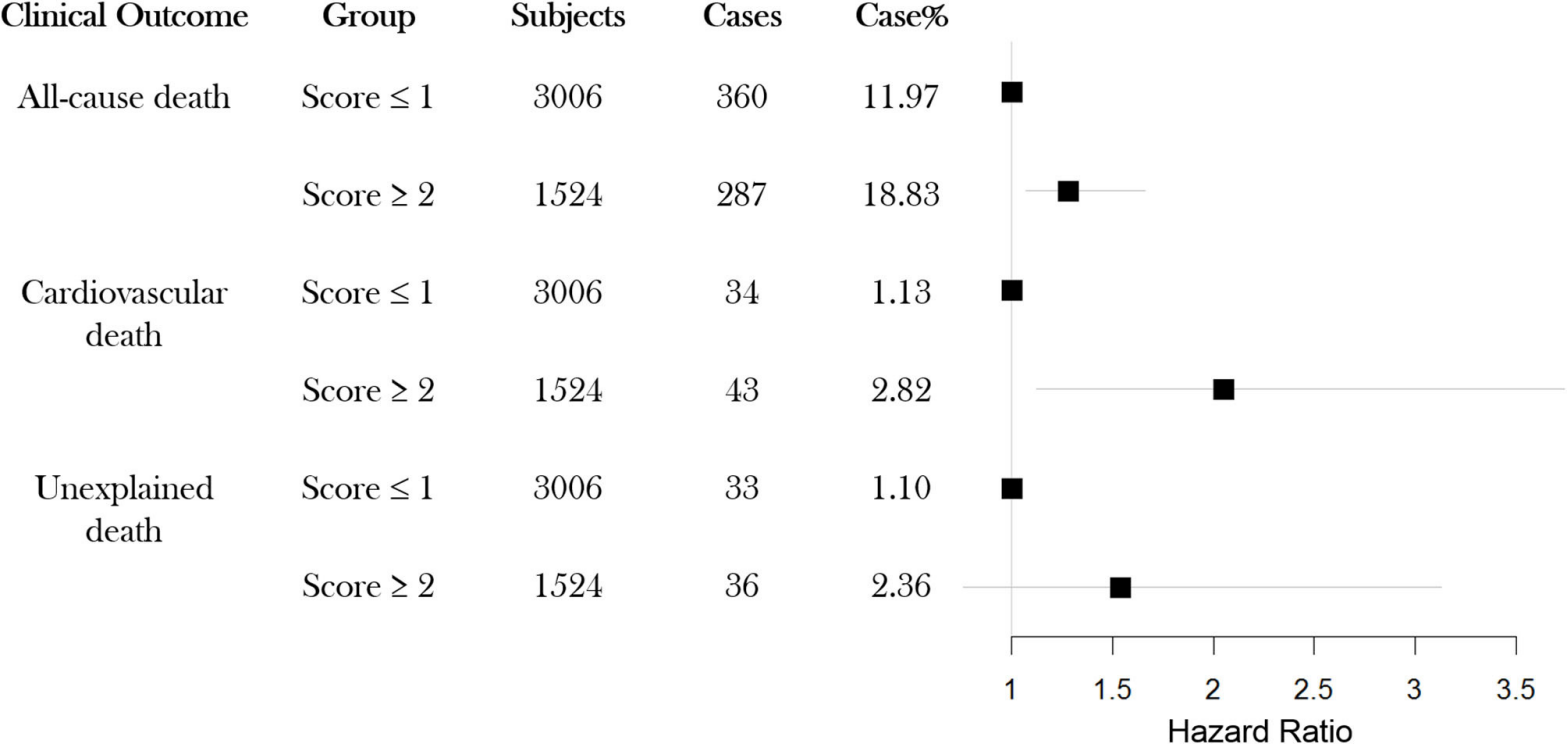
Event rates for each of three possible outcomes All-cause death, CV death and Unexpected death, for study subjects classified into two groups (high-EA or low-EA) based on ECG abnormal parameters (*N* = 4,530). EA: ECG abnormality; CV: cardiovascular.

To explore whether the EA score was associated with the survival outcomes, we examined the proportion of death events for each EA level. The results are illustrated in Supplementary Figure 2; the proportion of events for EA subgroups increased with each additional ECG abnormal parameter. Group with 3 ECG abnormalities exhibited the highest proportion of events for outcomes, all-cause death (10.25, 13.04, 18.39, and 19.82%), CV death (0.78, 129, 2.16, and 2.80%) and unexplained death (0.78, 1.29, 2.16, and 2.80%), respectively. We further performed a secondary analysis, using only two EA groups: low and high. The samples with no or one ECG abnormal parameter (0 or 1) were classified as low EA, whereas samples displaying two or more ECG abnormal parameters were classified as high EA. As shown in Figure 1, the high EA group showed a higher proportion of events for all-cause deaths (18.83%), CV deaths (2.82%) and unexplained deaths (2.36%) compared to that of the low EA group (11.97%, 1.13%, and 1.10%, respectively).

**Figure 2:**
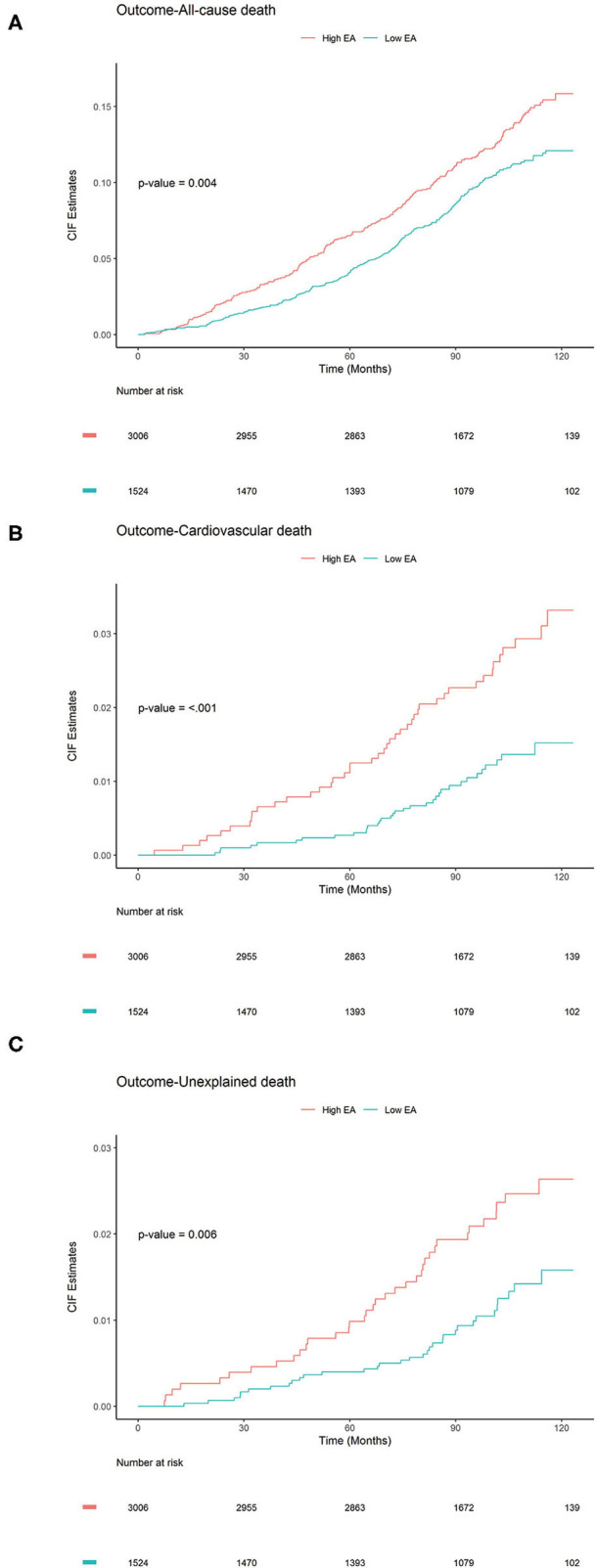
Cumulative Incidence Function plots to compare the cumulative incidence of subjects (*N* = 4,530) with ECG abnormality (EA) score (high, low). *P*-values were calculated using Gray's test to elucidate whether significant differences exist in the different groups: **(A)** All-cause death, **(B)** CV death, and **(C)** Unexplained death.

Furthermore, a competing risk survival analysis of the two-level EA groups were performed by using the Gray's tests to elucidate their associations with each of the death outcomes studied, while taking into consideration the possibility of the event to have an affect due to deaths by other causes (Figure 2). Notably, the high EA group showed significantly higher cumulative incidence in all three outcomes: all-cause death (*P* = 0.004, Figure 2A), CV death (*P* < 0.001, Figure 2B), and unexplained death (*P* = 0.006, Figure 2C). The detailed classification (using four EA categories) of each group and its association with the different death outcomes is shown in Supplementary Figure 3, which showed similar pattern as that of the two-level EA groups for all three events. In conclusion, these results indicate that ECG parameters can help in identifying people at a higher risk for all-cause-death, cardiovascular death, or unexplained death.

### Development of Prognostic Model

Supplementary Tables 5–13 displays the results for the scaled Schoenfeld residuals testing that were conducted for all three study outcomes to check for the validity of the proportional hazard assumption for the covariates under consideration, in each of the proposed prognostic models in this study. The findings along with the global tests were not found to be statistically significant, implying that the proportional hazard assumption is valid for all models. Cox - proportion hazards regression was performed on an age and sex adjusted univariate model and a multivariate adjusted prognostic model, to check if the EA score is a significant predictor for all three endpoints all-cause death, CV death and unexpected death. The results for the primary outcome, all-cause death are summarized in Table 3. Notably, the EA (high, low) was a significant predictor in the age- and sex-adjusted model (HR: 1.31; 95% CI= (1.12–1.53), *P* = 0.0008) and the multivariate model (HR: 1.28; 95% CI = (1.09–1.50); *P* = 0.002) after considering all traditional risk factors. The c-index was 0.786 in the multivariate model, suggesting a good-fit of the prediction model and the real death events in all-cause death. As shown in Supplementary Tables 14, 15, the EA score was a significant predictor for both CV death, and unexplained death, respectively.

**Table 3:** Prediction performance of ECG abnormality score (low EA and high EA) associated with all-cause death (*N* = 4,530).

**Event**	**Low**	**High**
	***N =*** **3,006**	***N =*** **1,524**
**All-cause death**		
^#^All-cause deaths (%)	360 (11.97)	287 (18.83)
Age-Adjusted and Sex-Adjusted HR (95%CI)	1	1.31 (1.12–1.53)
*P*-Value		0.0008
Multivariate-Adjusted HR (95% CI)	1	1.28 (1.09–1.50)
*P*-Value		0.002

# *The ECG abnormality score is a sum of ECG abnormality for each of 9 ECG parameters. The age-adjusted and sex-adjusted and the multivariate-adjusted HRs and 95% CIs. Deaths in the 10-year follow-up period were calculated using Cox-proportional Hazards Model. Variables included in the multivariate analysis consisted of age, sex, systolic blood pressure, diastolic blood pressure, BMI, smoking status, hypertension, diabetes mellitus, hyperlipidemia, and chronic kidney disease. Low EA is defined as < = 1 ECG abnormalities, and high EA is defined as > = 2 ECG abnormalities*.

### ECG Abnormality Score Validation and Model Improvement

To evaluate the performance of the multivariate adjusted prediction model in comparison with the traditional model, discrimination analysis was conducted using a 10CV design. Table 4, Supplementary Table 16 lists the average and the standard deviations of the C- indices over all 10CVs for both the models and for all three study outcomes. Harrel's c-index values for all three outcomes were reported to be 0.786, 0.798, and 0.851, implying that the ECG model is an effective predictor of the study events and can correctly discriminate patient's survival for a given patient pair. Furthermore, comparison of the c-indices between ECG model and traditional model, using a bootstrapping strategy, revealed that adding ECG parameters can significantly improve the predictive capability (*P* < 0.001), based on our data.

**Table 4:** Average and standard deviation of c-indices from 10-fold cross-validation.

**Models**	**All-cause death**		**CV death**		**Unexplained death**	
	**Avg_C-index**	**Stdev_C-index**	**Avg_C-index**	**Stdev_C-index**	**Avg_C-index**	**Stdev_C-index**
Multivariate adjusted ECG model	0.786	0.001	0.798	0.015	0.851	0.007
Traditional Model	0.775	0.001	0.782	0.016	0.846	0.007

*Multivariate adjusted model: using ECG abnormality (high, low) adjusted by age, sex, systolic blood pressure, diastolic blood pressure, BMI, current smoker, Dx_HTN, Dx_DM, Dx_HypLipid, Dx_CRD, Dx_CKD, and Dx_Stroke. Avg_C-index: average of c-indices over all 10 cross-validations for the training data. Stdev_C-index: standard deviation of c-indices over all 10 cross-validation for the training data*.

**Figure 3:**
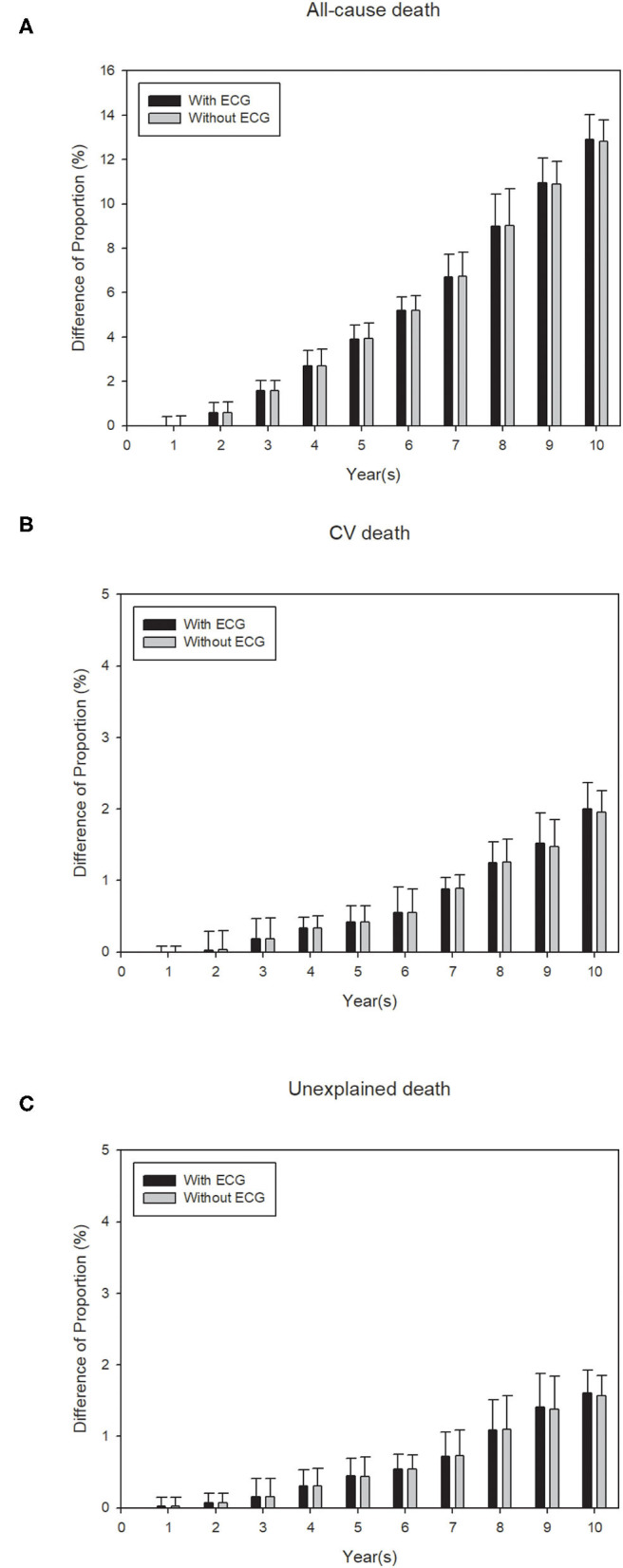
Calibration plots for the events **(A)** all-cause death, **(B)** cardiovascular death and, **(C)** unexplained death, to show the difference between observed and predicted survival probability for proposed ECG model (with EA score (high, low) and traditional variables) and traditional model (only traditional variables without EA score). Calibration for each of the models is conducted using 10-fold cross-validation (CV) and each bar shows an average of the probability difference over 10 models for each CV. Black bars: model with EA score + traditional variables (with ECG). Gray bars: model with only traditional variables (without ECG).

Finally, a calibration analysis was conducted to examine if the predicted events were significantly different from the observed events, for all three study outcomes. Figure 3 displays the results of the calibration analysis that was performed using a 10CV for each of the 10 years where the average difference of the proportions between the predicted and observed events were plotted for both ECG model (high, low) and traditional model. For all-cause death the differences in the proportions were ≤5% for the first 5 years, while for both CV death and unexpected death the differences in the proportions were negligible (<3%) for all 10 years. The higher differences that were observed for all-cause death after 5 years may be attributed to the fact that these elder patients may die of diseases other than cardiovascular ones, such as cancers. Similar patterns were observed for the other EA score model (0, 1, 2, and 3) (Supplementary Figure 4) and traditional models (Figure 3 and Supplementary Figure 4). Supplementary Tables 17–22 further lists the detailed calibration analysis results for both ECG model and traditional model, for all three outcomes. All results suggest that our prediction model can help to identify subjects with a high risk of a death event among the elderly.

## Discussion

Due to the increasing elderly population, world-wide, risk assessment and stratification of elderly people becomes an important issue in patient care. To our knowledge, this is the first study to specifically investigate the usefulness of an EA score as a predictive tool for an elderly population (mean age ≥65 years).

This study identified LVH, QTc and PR interval to be independently associated with increased risk of the primary study outcome, all-cause mortality, while LVH and PR as the only risk factor of cardiovascular mortality and unexplained mortality, respectively. Previous studies, including the Framingham experience, have demonstrated that LVH, manifested by repolarization abnormality and increased voltage, is one of the less common but ominous risk factors for coronary artery disease, stroke and heart failure. It was associated with a 3–15-fold increase of cardiovascular events (31–33). QTc and PR prolongation has also been reported as cardiac risk factors of sudden death (34, 35) in coronary patients or risk equivalent for middle age and older adults (36). Our findings were not only consistent with previous studies, but also extended the utility of the three ECG parameters to community-based elder individuals.

Several ECG parameters have been tested in many previous studies, both in general populations (37, 38) and in cohorts with cardiac specific diseases (39, 40). These studies showed that the predictive value of a single ECG parameter for sudden cardiac death was generally lower than combining multiple ECG parameters (6, 41, 42). This study uses a specific set of 9 ECG parameters to define the EA score, used to construct the prognostic models. This is because, these ECG parameters have more clinical meanings after having been used in medical practice for 100 years and their potential in predicting the outcomes of cardiovascular events have already been verified through numerous prior studies (6, 20, 21, 43–48) One of the more representative studies is one where the authors analyzed 6,830 participants and reported important ECG abnormalities associated with sudden cardiac death risks (6). To make the results in this study comparable to the previous study, the same ECG parameters were used for defining the EA score in this study. After adjusting for multiple traditional cardiovascular risk factors, our combined and validated EA scores successfully revealed that elderly subjects with ≥3 ECG abnormalities had a 29% higher risk of all-cause mortality compared to elderly subjects with ≤3 ECG abnormalities. Combined ECG risk parameters and traditional cardiovascular risk factors have a cumulative effect on the risk of all-cause mortality. In summary, these results suggest that the combined EA score has the potential to serve as a predictive tool of death events in the elderly.

ECG is a globally used, essential, inexpensive, and non-invasive technique to detect electric abnormalities of the heart and more and more elderly individuals receive annual health examinations, including ECG. We expect that ECG abnormalities would be an incidental finding during a routine health examination. Our study provides an important reference for clinicians or health professionals when they encounter asymptomatic elderly individuals susceptible to chronic diseases or with comorbidities.

Although we performed internal validation, a limitation of this study is that the findings have not been validated in a prospective, external cohort. However, as our cohort takes into consideration the complexity associated with diverse outcomes of cardiac related deaths in general elder population, we believe that our results are generalizable to other similar elder cohorts. Future studies will be conducted to validate the findings on independent cohorts. Secondly, as some of the covariates in our proposed model for the primary outcome, all-cause death (diabetes mellitus and stroke) could possibly have a have a non-proportional hazard effect (Supplementary Tables 5–7), further studies are needed for better modeling by conducting the possible interaction of these variables with time. Lastly, detailed clinical information such as echocardiographic assessment (e.g., left ventricular ejection fraction), coronary angiography, or medications are not available in the HALST database. For screening purposes in a large cohort, these information are seldom available, thus this study mimics the setting in which screening would normally take place.

## Conclusion

The findings in this study show that combined EA score is highly associated with an increased risk of all-cause mortality, CV mortality and unexplained mortality in an elderly population, which will be confirmed through future validation in independent cohorts. Use of this abnormality score may improve the risk stratification for elderly population in clinical practice.

## Data Availability Statement

The raw data supporting the conclusions of this article will be made available by the authors, without undue reservation.

## Ethics Statement

The studies involving human participants were reviewed and approved by this study was approved by the Ethics Committee of the National Health Research Institutes and conducted according to the principles of the Declaration of Helsinki. The patients/participants provided their written informed consent to participate in this study.

## Author Contributions

The study was conceptualization and designed by T-PL, S-FY, J-MJ, C-YC, and J-YC. Enrolled individuals, collected data, and laboratory work were done by CH, I-SC, I-CW, C-CH, T-YC, and W-TT. Data analysis and interpretation were done by T-PL, AC, K-CL, S-FY, J-MJ, C-YC, and J-YC. Resources and supervision was done by CH and J-MJ. Writing, reviewing, and editing the manuscript was done by T-PL, AC, K-CL, S-FY, J-YC, and J-MJ. All authors have read and approved the final version of the submitted manuscript.

## Funding

This study was mainly supported by the National Health Research Institutes of Taiwan (Grant Numbers: PH-101-SP-01, PH-102-SP-01, and PH-103-SP-01), partly by the Ministry of Science and Technology of Taiwan (Grant Numbers: MOST 106-2314-B-002-134-MY2, MOST 106-2314-B-002-206, MOST 107-2314-B-002-009, MOST 107-2314-B-002-261-MY3, and MOST 108-2314-B-002-187), and the Taiwan Health Foundation, and National Taiwan University Hospital (Grant Numbers: NTUH-UN105-012 and NTUH 106-018).

## Conflict of Interest

The authors declare that the research was conducted in the absence of any commercial or financial relationships that could be construed as a potential conflict of interest.

## Publisher's Note

All claims expressed in this article are solely those of the authors and do not necessarily represent those of their affiliated organizations, or those of the publisher, the editors and the reviewers. Any product that may be evaluated in this article, or claim that may be made by its manufacturer, is not guaranteed or endorsed by the publisher.
