# Supplementary material for: Develop and Apply Electrocardiography-Based Risk Score to Identify Community-Based Elderly Individuals at High-Risk of Mortality
Source: Front Cardiovasc Med. 2021 Oct 8;8:738061. doi: 10.3389/fcvm.2021.738061 (PMC8531436; doi:10.3389/fcvm.2021.738061)
Supplement: Supplementary file 1 [file Data_Sheet_1.PDF]

SUPPLEMENTARY MATERIAL

Develop and Apply Electrocardiography-based Risk Score to Identify Community-Based Elderly Individuals at High-Risk of Mortality

Tzu-Pin Lu[1,2], Amrita Chattopadhyay[1,2], Kuan-Chen Lu[1,2], Jing-Yuan Chuang[6], Shih-Fan Sherri Yeh[3], I-Shou Chang[4], Ching-Yu Julius Chen[5], I-Chien Wu[4], Chih-Cheng Hsu[4], Tzu-Yu Chen[4], Wei-Ting Tseng[4], Chao Agnes Hsiung[4], Jyh-Ming Jimmy Juang[5*]

[1] Department of Public Health, Institute of Epidemiology and Preventive Medicine, National Taiwan University, No. 17, Xu-Zhou Road, Taipei 10055, Taiwan

[2]Bioinformatics and Biostatistics Core, Center of Precision and Genomic Medicine, National Taiwan University, No. 2, Xu-Zhou Road, Taipei, 10002, Taiwan

[3] Department of Environmental and Occupational Medicine, National Taiwan University Hospital Hsin-Chu Branch, Hsin-Chu, Taiwan (No. 25, Lane 442, Sec. 1, Jingguo Rd, Hsinchu City 300, Taiwan)

[4]Institute of Population Health Sciences, National Health Research Institutes, Zhunan, Taiwan (35 Keyan Rd, Zhunan, Miaoli County 35053, Taiwan)

[5]Cardiovascular Center and Division of Cardiology, Department of Internal Medicine, National Taiwan University Hospital and National Taiwan University College of Medicine, Taipei, Taiwan (No. 7, Chung Shan S. Rd, Zhongzheng Dist., Taipei City 10002, Taiwan)

[6] Department of Medical Laboratory Science and Biotechnology, China Medical University, Taichung, Taiwan

*Correspondence and reprint requests to: Jyh-Ming Jimmy Juang, MD, PhD, Cardiovascular Center and Division of Cardiology, Department of Internal Medicine, National Taiwan University Hospital, No. 7, Chung-Shan South Road, Taipei 100, Taiwan

Tel: 886-2-23562209; Fax: 886-2-23941938; E-mail: jjmjuang@ntu.edu.tw

1. Supplementary materials

Study subjects: Taiwan Geriatric Health Survey population

Taiwan is an island with a population of approximately 23.5 million people. Their ancestors moved to Taiwan from southeastern China about 400–500 years ago, and no major population immigration or admixture has subsequently occurred. Thus, the majority (>95%) of Taiwanese are of Han Chinese ancestry, whereas ~2% are of aboriginal ancestry (Austronesian). We performed a prospective community-based cohort study in Taiwan (Ongoing since December 2008, the Healthy Aging Longitudinal Study in Taiwan, or HALST study, is a community-based cohort study), that enrolled 5,380 subjects. Each volunteer provided signed informed consent, and this study was approved by the Ethics Committee of the National Health Research Institutes and adhered to the principles outlined in the Declaration of Helsinki. Taiwanese aborigines were not included. In brief, a representative random sample of the entire national population was selected by citizen ID from seven communities in northern, central, southern, and eastern regions of Taiwan. Eligible and willing participants were enrolled from December 2008 to March 2013. All participants were prospectively followed on a regular basis within the framework of HALST, and follow-up information was available until April 2019. In case of death, death certificates were obtained from the National Taiwan Ministry of Health and Welfare and evaluated using the 10th revision of the International Classification of Diseases (ICD-10).

2. **Supplementary Tables**

Table S1. Demographic data and clinical characteristics of the study population.

**Characteristic**		**Measurement (N=4,530)**
Male, n (%)		2,145 (47.4%)
Female, n (%)		2,385 (52.6%)
Age at enrollment (years)		69.04±8.14
Body mass index (kg/m^2)		24.5±3.5
Systolic BP (mmHg)		128.4±18.7
Diastolic BP (mmHg)		70.4±10.6
Smoking	Current	591 (13.1%)
	Former	695 (15.3%)
	Never	3,244 (71.6%)
Essential hypertension, n (%)		1,972 (43.5%)
Diabetes mellitus, n (%)		804 (17.7%)
Stroke, n (%)		220 (4.9%)
Hyperlipidemia, n (%)		1,442 (31.8%)
Chronic kidney disease, n (%)		658 (14.5%)
PR interval, *ms*		168.8±26.3
QRS duration, *ms*		94.3±14.06
QRS-T angle, degree		28.06±36.01
QTc*, ms		436.9±22.5

QTc is calculated by Bazett’s equations. BP: blood pressure

Table S2. Association between ECG parameters and cardiovascular death#

		Univariate model		Multivariate model	
**ECG Parameters**	**n(%)**	**HR (95%CI)**	**P value**	**HR (95%CI)**	**P value**
Heart rate > 80/min	452 (9.97)	1.14 (0.59 - 2.23)	0.693	1.07 (0.54 - 2.12)	0.84
Early repolarization pattern (ERP)	880 (19.42)	1.17 (0.66 - 2.07)	0.582	1.13 (0.63 - 2.02)	0.69
Fragmented QRS complex (fQRS)	1869 (41.26)	0.99 (0.64 - 1.57)	0.987	0.99 (0.62 - 1.56)	0.96
ST elevation in lead aVR	0 (0)	NA	NA	NA	NA
Left ventricular hypertrophy (LVH)*	735 (16.22)	2.37 (1.47 - 3.78)	0.000291	2.37 (1.48 - 3.79)	0.0003
QRS duration > 110 ms	311 (6.86)	1.22 (0.58 - 2.58)	0.598	1.05 (0.49 - 2.29)	0.89
Corrected QT interval (QTc) (ms)	1001 (22.09)	1.52 (0.90 - 2.56)	0.114	1.46 (0.85 - 2.52)	0.17
PR interval (ms)	115 (2.54)	1.25 (0.45 - 3.44)	0.672	1.23 (0.45 - 3.41)	0.69
QRS-T angle >90 degrees	88 (1.94)	1.49 (0.36 - 6.07)	0.579	1.63 (0.39 - 6.75)	0.5

# P-value (age-adjusted and sex-adjusted) is calculated from a Cox hazard regression model. HR: hazard ratio; CI: confidence interval; * P value <0.05

Table S3. Association between ECG parameters and unexplained death.[#]

		Univariate model		Multivariate model	
**ECG Parameter**	**n(%)**	**HR (95%CI)**	**P value**	**HR (95%CI)**	**P value**
Heart rate > 80/min	452 (9.97)	1.52 (0.81 - 2.85)	0.19	1.37 (0.72 - 2.62)	0.34
Early repolarization pattern (ERP)	880 (19.42)	0.97 (0.51 - 1.85)	0.92	0.99 (0.51 - 1.93)	0.98
Fragmented QRS complex (fQRS)	1869 (41.26)	0.96 (0.59 - 1.54)	0.86	0.94 (0.58 - 1.54)	0.82
ST elevation in lead aVR	0 (0)	NA	NA	NA	NA
Left ventricular hypertrophy (LVH)	735 (16.22)	1.51 (0.89 - 2.56)	0.13	1.51 (0.88 - 2.57)	0.13
QRS duration > 110 ms	311 (6.86)	1.66 (0.87 - 3.31)	0.15	1.50 (0.73 - 3.08)	0.27
Corrected QT interval (QTc) (ms)	1001 (22.09)	1.67 (0.97 - 2.87)	0.07	1.45 (0.81 - 2.58)	0.21
PR interval (ms)*	50 (2.2)	2.63 (1.24 - 5.56)	0.01	2.63 (1.24 - 5.57)	0.01
QRS-T angle >90 degrees	88 (1.94)	0.84 (0.17 - 6.07)	0.86	0.77 (0.11 - 5.65)	0.79

[#] P-value(age-adjusted and sex-adjusted) is calculated from a Cox hazard regression model. HR: hazard ratio; CI: confidence interval; * P value <0.05

Table S4. P- values for all possible 2- group comparisons for EA (0,1,2,3)

Demographic Factors	EA score groups (comparison P-values)					
	**0 -1**	**0 - 2**	**0 - 3**	**1 -2**	**1 - 3**	**2 - 3**
**Male (%)**	0.36	0.82	0.32	0.32	0.11	0.32
**Age (years)**	0.51	$8.6x10^{-5}$	$4.3x10^{-5}$	$3.1x10^{-3}$	$9.9x10^{-4}$	0.681
**Systolic blood pressure (mmHg)**	0.99	0	0	0	0	0.009
**Diastolic blood pressure (mmHg)**	0.9	0.006	$1x10^{-6}$	$1x10^{-4}$	$1x10^{-7}$	0.038
**Body Mass index**	0.91	0.86	0.73	0.99	0.92	0.98
**Current Smoker (%)**	0.015	0.39	0.46	0.39	0.39	0.47
**Hypertension (%)**	0.71	0	0	0	0	0.004
**Diabetes Mellitus (%)**	0.34	0.34	0.19	0.82	0.34	0.34
**Stroke(%)**	0.69	0.36	0.36	0.36	0.36	0.87
**Hyperlipidemia (%)**	0.81	0.81	0.87	0.81	0.87	0.81
**Chronic respiratory disease (%)**	0.47	0.47	0.47	1	1	1
**Chronic kidney disease (%)**	0.85	0.75	0.85	0.75	0.75	0.91

$P<0.05$ is the threshold for significance, EA: ECG abnormality

Table S5. Cox-proportional hazards assumption checks for ECG model with EA score# (0,1,2,3) for outcome all-cause death

Model parameters	chisq	df	p
EA score	2.78	1	0.095
Diastolic blood pressure (mmHg)	1.99	1	0.158
Systolic blood pressure (mmHg)	0.19	1	0.661
Body Mass index	1.50	1	0.221
Smoking status	0.03	1	0.860
Hypertension	2.18	1	0.139
Diabetes Mellitus	3.72	1	0.054
Hyperlipidemia	0.74	1	0.389
Chronic respiratory disease	0.28	1	0.599
Chronic kidney disease	0.41	1	0.522
Stroke	3.95	1	0.047
Gender	0.00	1	0.989
Age	0.00	1	0.951
**GLOBAL**	**26.63**	**13**	**0.014**

EA: ECG abnormality

Table S6. Cox-proportional hazards assumption checks for ECG model with EA# (high, low) for outcome all-cause death

Model Parameters	chisq	df	p
**EA**	2.33	1	0.127
**Diastolic blood pressure (mmHg)**	1.94	1	0.164
**Systolic blood pressure (mmHg)**	0.18	1	0.674
**Body Mass index**	1.58	1	0.209
**Smoking status**	0.04	1	0.852
**Hypertension**	2.23	1	0.135
**Diabetes Mellitus**	3.81	1	0.051
**Hyperlipidemia**	0.72	1	0.396
**Chronic respiratory disease**	0.27	1	0.606
**Chronic kidney disease**	0.43	1	0.511
**Stroke**	4.23	1	0.040
**Gender**	0.00	1	0.971
**Age**	0.00	1	0.963
**GLOBAL**	**26.47**	**13**	**0.015**

EA: ECG abnormality

Table S7. Cox-proportional hazards assumption checks for traditional model for outcome all-cause death

Model Parameters	chisq	df	p
**Diastolic blood pressure (mmHg)**	1.94	1	0.164
**Systolic blood pressure (mmHg)**	0.16	1	0.692
**Body Mass index**	1.50	1	0.221
**Smoking status**	0.02	1	0.899
**Hypertension**	2.21	1	0.137
**Diabetes Mellitus**	3.91	1	0.048
**Hyperlipidemia**	0.74	1	0.389
**Chronic respiratory disease**	0.27	1	0.605
**Chronic kidney disease**	0.40	1	0.529
**Stroke**	4.11	1	0.043
**Gender**	0.00	1	0.977
**Age**	0.01	1	0.940
**GLOBAL**	**23.84**	**12**	**0.021**

Table S8. Cox-proportional hazards assumption checks for ECG model with EA# score (0,1,2,3) for outcome CV death

Model Parameters	chisq	df	p
**EA score**	3.82	1	0.051
**Diastolic blood pressure (mmHg)**	1.29	1	0.256
**Systolic blood pressure (mmHg)**	0.00	1	0.966
**Body Mass index**	0.05	1	0.819
**Smoking status**	0.37	1	0.545
**Hypertension**	0.20	1	0.654
**Diabetes Mellitus**	0.60	1	0.437
**Hyperlipidemia**	0.61	1	0.435
**Chronic respiratory disease**	0.00	1	0.952
**Chronic kidney disease**	0.65	1	0.419
**Stroke**	0.04	1	0.835
**Gender**	0.01	1	0.941
**Age**	0.00	1	0.999
**GLOBAL**	**10.15**	**13**	**0.681**

EA: ECG abnormality

Table S9. Cox-proportional hazards assumption checks for ECG model with EA# (high, low) for outcome CV death

Model Parameters	chisq	df	p
**EA**	1.16	1	0.280
**Diastolic blood pressure (mmHg)**	1.27	1	0.256
**Systolic blood pressure (mmHg)**	0.00	1	0.980
**Body Mass index**	0.04	1	0.842
**Smoking status**	0.38	1	0.544
**Hypertension**	0.21	1	0.643
**Diabetes Mellitus**	0.62	1	0.430
**Hyperlipidemia**	0.60	1	0.440
**Chronic respiratory disease**	0.00	1	0.950
**Chronic kidney disease**	0.69	1	0.400
**Stroke**	0.07	1	0.790
**Gender**	0.00	1	0.962
**Age**	0.00	1	1.000
**GLOBAL**	**7.36**	**13**	**0.884**

EA: ECG abnormality

Table S10. Cox-proportional hazards assumption checks for traditional model for outcome CV death

Model Parameters	chisq	df	p
**Diastolic blood pressure (mmHg)**	1.23	1	0.2669
**Systolic blood pressure (mmHg)**	0.00	1	0.9829
**Body Mass index**	0.05	1	0.8210
**Smoking status**	0.31	1	0.5762
**Hypertension**	0.23	1	0.6334
**Diabetes Mellitus**	0.68	1	0.4106
**Hyperlipidemia**	0.61	1	0.4346
**Chronic respiratory disease**	0.00	1	0.9580
**Chronic kidney disease**	0.64	1	0.4228
**Stroke**	0.05	1	0.8159
**Gender**	0.01	1	0.9303
**Age**	0.00	1	0.9752
**GLOBAL**	**6.05**	**12**	**0.9138**

Table S11. Cox-proportional hazards assumption checks for ECG model with EA# score (0,1,2,3) for outcome unexplained death

Model Parameters	chisq	df	p
**EA score**	1.98	1	0.1593
**Diastolic blood pressure (mmHg)**	0.07	1	0.7872
**Systolic blood pressure (mmHg)**	1.04	1	0.3068
**Body Mass index**	1.06	1	0.3028
**Smoking status**	0.40	1	0.5258
**Hypertension**	1.11	1	0.2915
**Diabetes Mellitus**	0.03	1	0.8647
**Hyperlipidemia**	0.11	1	0.7410
**Chronic respiratory disease**	0.23	1	0.6315
**Chronic kidney disease**	0.55	1	0.4603
**Stroke**	1.20	1	0.2738
**Gender**	0.11	1	0.7441
**Age**	0.37	1	0.5416
**GLOBAL**	**6.73**	**13**	**0.9157**

EA: ECG abnormality

Table S12. Cox-proportional hazards assumption checks for ECG model with EA[#] (high, low) for outcome CV death

Model Parameters	chisq	df	p
**EA**	2.26	1	0.130
**Diastolic blood pressure (mmHg)**	0.08	1	0.770
**Systolic blood pressure (mmHg)**	1.07	1	0.304
**Body Mass index**	1.15	1	0.282
**Smoking status**	0.39	1	0.530
**Hypertension**	1.14	1	0.290
**Diabetes Mellitus**	0.02	1	0.890
**Hyperlipidemia**	0.11	1	0.740
**Chronic respiratory disease**	0.24	1	0.630
**Chronic kidney disease**	0.56	1	0.450
**Stroke**	1.34	1	0.250
**Gender**	0.12	1	0.730
**Age**	0.36	1	0.551
**GLOBAL**	**7.15**	**13**	**0.891**

EA: ECG abnormality

Table S13. Cox-proportional hazards assumption checks for traditional model for outcome CV death

Model Parameters	chisq	df	p
**Diastolic blood pressure (mmHg)**	0.10	1	0.7515
**Systolic blood pressure (mmHg)**	1.22	1	0.2691
**Body Mass index**	1.17	1	0.2793
**Smoking status**	0.44	1	0.5087
**Hypertension**	1.22	1	0.2696
**Diabetes Mellitus**	0.01	1	0.9217
**Hyperlipidemia**	0.12	1	0.7285
**Chronic respiratory disease**	0.25	1	0.6166
**Chronic kidney disease**	0.49	1	0.4827
**Stroke**	1.30	1	0.2537
**Gender**	0.13	1	0.7180
**Age**	0.39	1	0.5346
**GLOBAL**	5.43	12	0.9420

Table S14. Prediction performance of EA# in the validation set associated with CV death.

Event	Low<br>n=3006	High<br>n=1524
**Cardiovascular death**		
#Cardiovascular deaths (%)	34 (1.13)	43 (2.82)
Age-Adjusted and Sex-Adjusted HR (95%CI)	1	2.00 (1.27 - 3.15)
P-Value		0.002
Multivariate-Adjusted HR (95% CI)	1	1.96 (1.24 - 3.09)
P-Value		0.004

The EA score consist of 9 ECG abnormalities. The Age-adjusted and Sex-adjusted and the multivariate adjusted HRs and 95% CIs in the 10-year follow up were calculated using Cox-proportional Hazards Model. Variables included in the multivariate analysis consisted of age, sex, systolic Blood Pressure, Diastolic Blood Pressure, Body Mass Index, Current smoker, Dx_HTN, Dx_DM, Dx_HypLipid, Dx_CRD, Dx_CKD, and Dx_Stroke. Low EA is defined as EA scores with <= 1 ECG abnormalities and high EA is defined as EA scores with >=2 ECG abnormalities. # EA: ECG abnormality

Table S15. Prediction performances of EA# score in the validation set associated with unexplained death.

Event	Low<br>n=3006	High<br>n=1524
**Unexplained death**		
#Unexplained deaths	33	36
Age-Adjusted and Sex-Adjusted HR (95%CI)	1	1.63 (1.01- 2.61)
P-Value		0.04
Multivariate-Adjusted HR (95% CI)	1	1.66 (1.02 - 2.69)
P-Value		0.04

The EA score consist of 9 ECG abnormalities. The Age-adjusted and Sex-adjusted and the multivariate adjusted HRs and 95% CIs in the 10-year follow up were calculated using Cox-proportional Hazards Model. Variables included in the multivariate analysis consisted of age, sex, systolic Blood Pressure, Diastolic Blood Pressure, Body Mass Index, Current smoker, Dx_HTN, Dx_DM, Dx_HypLipid, Dx_CRD, Dx_CKD, and Dx_Stroke. Low EA is defined as EA scores with <= 1 ECG abnormalities and high EA is defined as EA scores with >=2 ECG abnormalities. # EA: ECG abnormality

Table S16: Average and standard deviation of C-Indices from 10-fold cross-validation

Models	All- cause death		CV death		Unexplained death	
	Avg_C-index	Stdev_C-Index	Avg_C-index	Stdev_C-Index	Avg_C-index	Stdev_C-Index
**Multivariate adjusted ECG model**	0.781	0.002	0.795	0.016	0.852	0.007
**Traditional Model**	0.775	0.001	0.782	0.016	0.846	0.007

Multivariate adjusted model: using EA score (0,1,2,3) adjusted by age, sex, systolic Blood Pressure, Diastolic Blood Pressure, Body Mass Index, Current smoker, Dx_HTN, Dx_DM, Dx_HypLipid, Dx_CRD, Dx_CKD, and Dx_Stroke. Avg_C-index: average of c-indices over all 10 cross-validations for the training data. Stdev_C-index: standard deviation of c-indices over all 10 cross-validation for the training data

Table S17. Calibration analysis results using a 10-fold internal cross-validation with EA# (high, low) and traditional variables for event All-cause death

Year	p_diff	p_diff	p_diff	p_diff	p_diff	p_diff	p_diff	p_diff	p_diff	p_diff	average_p_diff	stdev_p_diff
**1**	0.66%	-0.52%	-0.08%	-0.32%	-0.55%	0.23%	0.30%	0.14%	-0.03%	0.38%	0.02%	0.003
**2**	1.18%	0.69%	0.55%	0.08%	-0.16%	0.77%	1.05%	0.80%	0.93%	0.12%	0.60%	0.004
**3**	1.72%	2.41%	1.53%	1.14%	1.67%	2.19%	1.38%	1.42%	1.49%	0.97%	1.59%	0.004
**4**	2.86%	2.76%	3.69%	2.51%	2.14%	3.23%	3.22%	1.75%	1.65%	3.18%	2.70%	0.006
**5**	3.57%	3.80%	3.13%	3.41%	3.90%	4.83%	3.61%	5.09%	4%	3.86%	3.92%	0.006
**6**	5.35%	5.30%	3.81%	5.14%	5.65%	6.11%	5.19%	5.48%	4.49%	5.42%	5.19%	0.006
**7**	7.29%	7.23%	4.26%	7.81%	5.83%	6.68%	7.32%	7.74%	6.48%	6.44%	6.71%	0.01
**8**	8.71%	8.38%	7.15%	7.04%	9.15%	11.67%	10.34%	10.29%	8.16%	9.11%	9.00%	0.01
**9**	12.49%	10.34%	9.39%	9.66%	11.14%	NaN%	NaN%	11.01%	11.47%	12.48%	11.00%	0.01
**10**	12.94%	13.05%	12.61%	11.62%	14.58%	NaN%	NaN%	NaN%	NaN%	NaN%	12.96%	0.01

EA: ECG abnormality

Table S18. Calibration analysis results using a 10-fold internal cross-validation using traditional variables only for event All-cause death

Year	p_diff	p_diff	p_diff	p_diff	p_diff	p_diff	p_diff	p_diff	p_diff	p_diff	average_p_diff	stdev_p_diff
**1**	0.63%	-0.53%	-0.07%	-0.33%	-0.57%	0.25%	0.33%	0.16%	-0.02%	0.36%	0.02%	0.004
**2**	1.08%	0.64%	0.59%	0.04%	-0.22%	0.82%	1.15%	0.84%	0.94%	0.09%	0.60%	0.005
**3**	1.56%	2.34%	1.59%	1.07%	1.58%	2.30%	1.56%	1.50%	1.50%	0.91%	1.59%	0.004
**4**	2.63%	2.66%	3.79%	2.40%	2%	3.39%	3.49%	1.86%	1.65%	3.10%	2.70%	0.007
**5**	3.29%	3.65%	3.26%	3.27%	3.73%	5.05%	3.96%	5.23%	4%	3.75%	3.92%	0.007
**6**	4.99%	5.11%	3.95%	4.97%	5.42%	6.40%	5.65%	5.68%	4.49%	5.29%	5.20%	0.007
**7**	6.86%	6.99%	4.45%	7.63%	5.55%	7.04%	7.89%	8%	6.48%	6.31%	6.72%	0.01
**8**	8.25%	8.14%	7.37%	6.85%	8.88%	12.18%	10.87%	10.44%	8.23%	8.98%	9.02%	0.017
**9**	11.99%	10.12%	9.68%	9.49%	10.73%	NaN%	NaN%	11.17%	11.59%	12.27%	10.88%	0.011
**10**	12.44%	12.85%	13.07%	11.50%	14.17%	NaN%	NaN%	NaN%	NaN%	NaN%	12.81%	0.009

Table S19. Calibration analysis results using a 10-fold internal cross-validation with EA# (high, low) and traditional variables for event cardiovascular death

Year	p_diff	p_diff	p_diff	p_diff	p_diff	p_diff	p_diff	p_diff	p_diff	p_diff	average_p_diff	stdev_p_diff
**1**	0.03%	0.03%	-0.22%	0.03%	0.03%	0.03%	0.02%	0.02%	0.03%	0.02%	0.00%	0.0007
**2**	-0.53%	-0.03%	-0.33%	0.21%	0.23%	0.21%	0.13%	0.20%	0.21%	-0.05%	0.03%	0.002
**3**	-0.18%	0.38%	0.29%	0.39%	0.16%	0.39%	0.01%	0.37%	0.40%	-0.40%	0.18%	0.002
**4**	0.37%	0.50%	0.38%	0.24%	-0.01%	0.25%	0.28%	0.46%	0.48%	0.36%	0.33%	0.002
**5**	0.24%	0.65%	0.27%	0.68%	0.59%	0.62%	0.14%	0.61%	0.12%	0.26%	0.42%	0.002
**6**	0.58%	1.10%	0.08%	0.73%	1.02%	0.77%	0.13%	0.49%	0.46%	0.11%	0.55%	0.004
**7**	0.93%	0.64%	0.79%	1.03%	1.16%	1.07%	0.82%	0.76%	0.73%	0.89%	0.88%	0.002
**8**	1.32%	1.39%	0.78%	1.12%	1.51%	1.61%	1.08%	1.46%	0.73%	1.46%	1.25%	0.003
**9**	2.13%	2.14%	0.84%	1.41%	1.37%	NaN%	NaN%	1.25%	1.63%	1.35%	1.52%	0.004
**10**	2.60%	2.07%	1.62%	2.03%	1.73%	NaN%	NaN%	NaN%	NaN%	NaN%	2.01%	0.004

EA: ECG abnormality

Table S20. Calibration analysis results using a 10-fold internal cross-validation using traditional variables only for event cardiovascular death

Year	p_diff	p_diff	p_diff	p_diff	p_diff	p_diff	p_diff	p_diff	p_diff	p_diff	average_p_diff	stdev_p_diff
**1**	0.02%	0.03%	-0.22%	0.02%	0.03%	0.03%	0.02%	0.03%	0.03%	0.02%	0.00%	0.0007
**2**	-0.55%	-0.04%	-0.32%	0.20%	0.21%	0.23%	0.17%	0.22%	0.22%	-0.05%	0.03%	0.002
**3**	-0.21%	0.36%	0.30%	0.37%	0.13%	0.42%	0.07%	0.41%	0.41%	-0.42%	0.18%	0.003
**4**	0.33%	0.47%	0.40%	0.21%	-0.05%	0.29%	0.36%	0.51%	0.48%	0.35%	0.34%	0.002
**5**	0.19%	0.61%	0.30%	0.62%	0.54%	0.67%	0.25%	0.67%	0.12%	0.24%	0.42%	0.002
**6**	0.50%	1.03%	0.12%	0.65%	0.94%	0.86%	0.31%	0.59%	0.46%	0.08%	0.55%	0.003
**7**	0.81%	0.54%	0.86%	0.95%	1.05%	1.18%	1.06%	0.89%	0.72%	0.86%	0.89%	0.002
**8**	1.18%	1.29%	0.86%	1.03%	1.40%	1.75%	1.33%	1.60%	0.74%	1.43%	1.26%	0.003
**9**	1.95%	2.06%	0.94%	1.32%	1.21%	NaN%	NaN%	1.37%	1.65%	1.28%	1.47%	0.003
**10**	2.40%	2.01%	1.80%	1.97%	1.57%	NaN%	NaN%	NaN%	NaN%	NaN%	1.95%	0.003

Table S21. Calibration analysis results using a 10-fold internal cross-validation with EA[#] (high, low) and traditional variables for event unexplained death

Year	p_diff	p_diff	p_diff	p_diff	p_diff	p_diff	p_diff	p_diff	p_diff	p_diff	average_p_diff	stdev_p_diff
**1**	0.11%	0.09%	0.10%	-0.14%	0.10%	0.11%	0.08%	0.10%	-0.14%	-0.17%	0.02%	0.001
**2**	0.20%	0.16%	0.17%	-0.09%	-0.07%	0.19%	0.14%	-0.07%	0.15%	-0.10%	0.07%	0.001
**3**	0.37%	0.03%	0.07%	-0.48%	0.31%	0.36%	0.02%	0.30%	0.31%	0.27%	0.16%	0.003
**4**	0.35%	0.23%	0.53%	0.46%	-0.24%	0.37%	0.38%	0.52%	0.21%	0.23%	0.30%	0.002
**5**	0.14%	0.53%	0.62%	0.29%	-0.05%	0.70%	0.47%	0.63%	0.54%	0.56%	0.44%	0.002
**6**	0.77%	0.73%	0.27%	0.16%	0.61%	0.40%	0.63%	0.58%	0.47%	0.77%	0.54%	0.002
**7**	0.91%	0.54%	0.17%	1.03%	0.17%	0.99%	0.65%	1.17%	0.76%	0.83%	0.72%	0.003
**8**	1.27%	0.72%	0.34%	0.61%	0.94%	1.68%	1.33%	1.44%	1.34%	1.36%	1.10%	0.004
**9**	2.08%	0.71%	0.96%	1.15%	1.83%	NaN%	NaN%	1.75%	1.09%	1.81%	1.42%	0.005
**10**	1.96%	1.25%	1.37%	1.57%	2.02%	NaN%	NaN%	NaN%	NaN%	NaN%	1.63%	0.003

EA: ECG abnormality

Table S22. Calibration analysis results using a 10-fold internal cross-validation using traditional variables only for event unexpected death

Year	p_diff	p_diff	p_diff	p_diff	p_diff	p_diff	p_diff	p_diff	p_diff	p_diff	average_p_diff	stdev_p_diff
**1**	0.09%	0.08%	0.10%	-0.15%	0.10%	0.12%	0.09%	-0.14%	0.11%	-0.18%	0.02%	0.001
**2**	0.17%	0.14%	0.17%	-0.10%	-0.08%	0.20%	0.16%	0.17%	-0.06%	-0.11%	0.07%	0.001
**3**	0.31%	0.02%	0.08%	-0.49%	0.29%	0.37%	0.06%	0.33%	0.32%	0.25%	0.15%	0.002
**4**	0.26%	0.20%	0.55%	0.43%	-0.27%	0.41%	0.46%	0.21%	0.57%	0.20%	0.30%	0.002
**5**	0.04%	0.49%	0.64%	0.26%	-0.07%	0.75%	0.56%	0.53%	0.68%	0.52%	0.44%	0.002
**6**	0.63%	0.68%	0.29%	0.13%	0.58%	0.46%	0.77%	0.47%	0.65%	0.72%	0.54%	0.002
**7**	0.72%	0.46%	0.23%	1%	0.14%	1.08%	0.83%	0.75%	1.29%	0.78%	0.73%	0.003
**8**	1.03%	0.64%	0.39%	0.57%	0.90%	1.76%	1.45%	1.35%	1.61%	1.31%	1.10%	0.005
**9**	1.77%	0.63%	1.04%	1.12%	1.76%	NaN%	NaN%	1.11%	1.89%	1.72%	1.38%	0.005
**10**	1.65%	1.19%	1.49%	1.57%	1.96%	NaN%	NaN%	NaN%	NaN%	NaN%	1.57%	0.003

3. Supplementary Figures

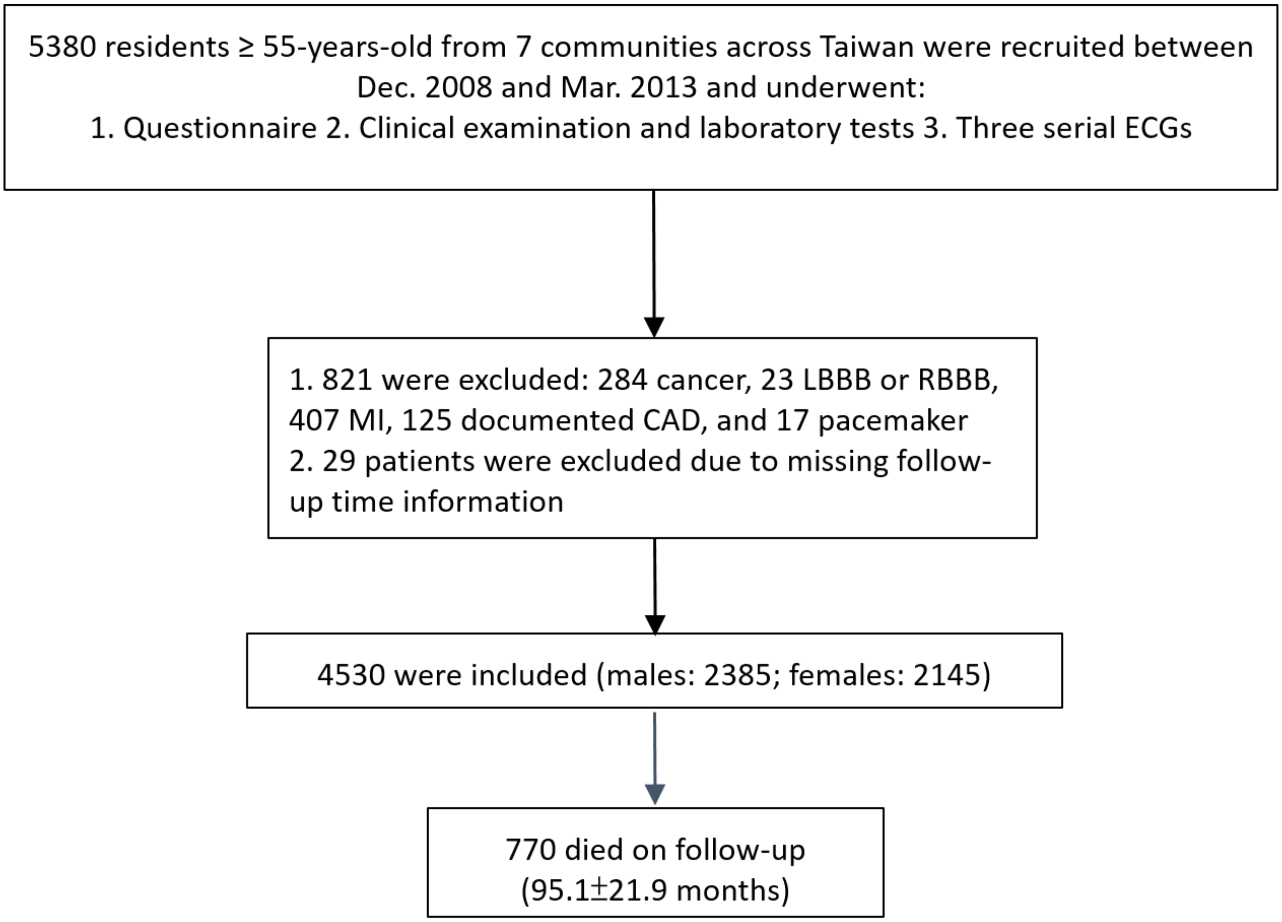

Figure S1. Flowchart of the enrollment of the HALST cohort

Clinical Outcomes	Group	Subjects	Cases	Case%
All-cause death	Score = 0	1151	118	10.25
	Score = 1	1855	242	13.04
	Score = 2	1060	195	18.39
	Score ≥ 3	464	92	19.82
Cardiovascular death	Score = 0	1151	9	0.78
	Score = 1	1855	25	1.34
	Score = 2	1060	32	3.02
	Score ≥ 3	464	11	2.37
Unexplained Death	Score =0	1151	9	0.78
	Score = 1	1855	24	1.29
	Score = 2	1060	23	2.16
	Score ≥ 3	464	13	2.80

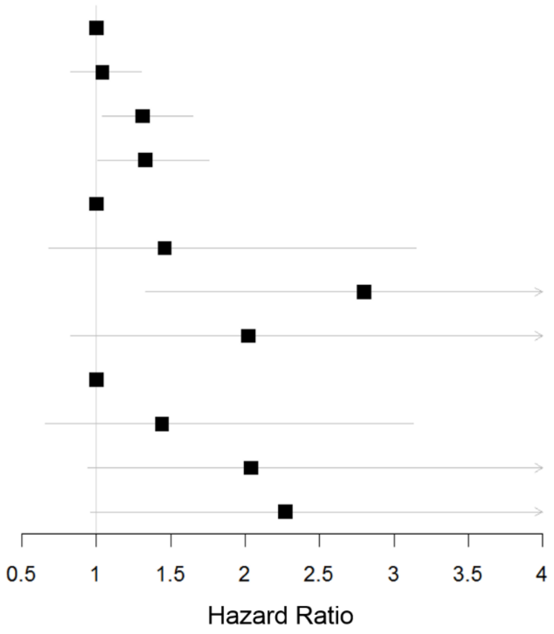

Figure S2. Event rates for each of three possible outcomes, broken down by four groups, with each group having a distinct number of ECG abnormality parameters

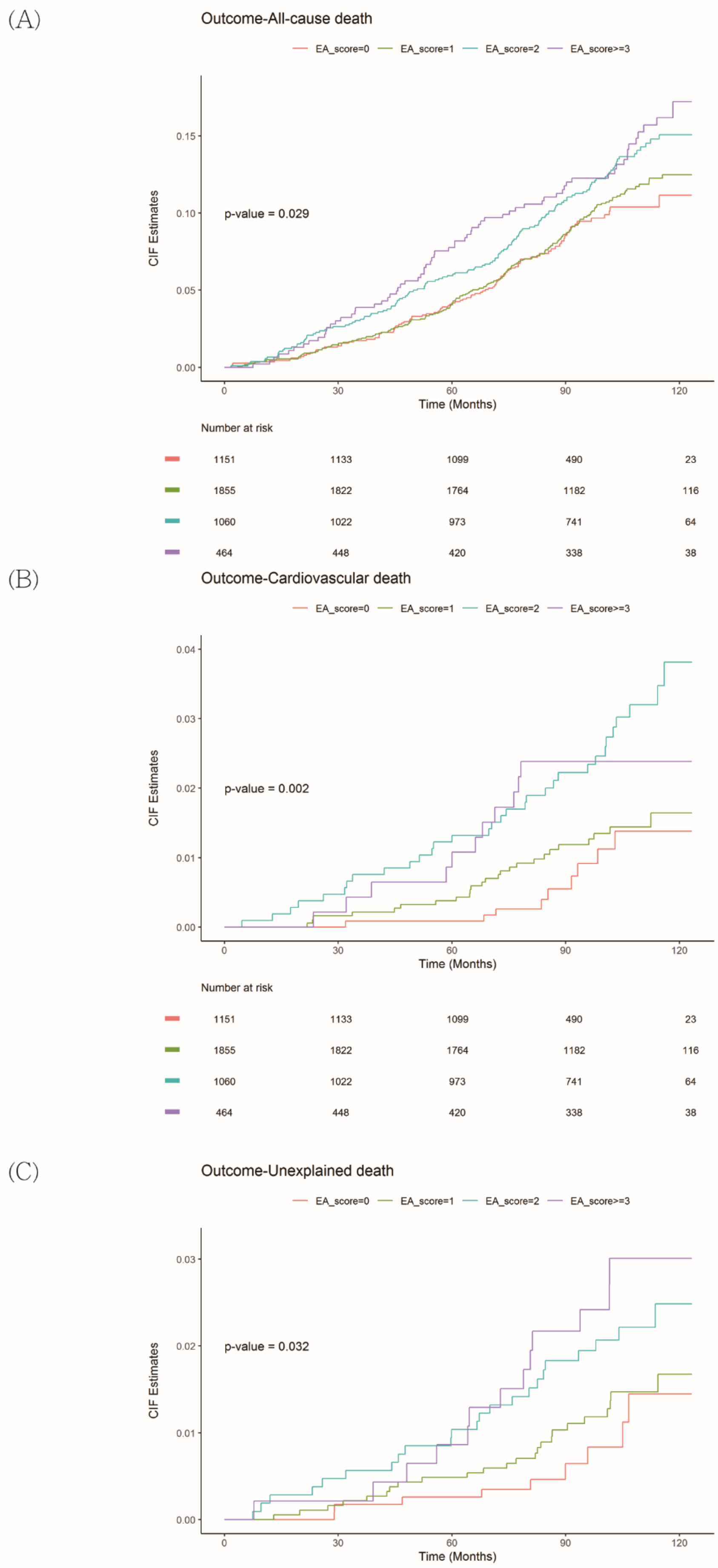
(A)
Outcome-All-cause death
EA_score=0
EA_score=1
EA_score=2
EA_score>=3
p-value = 0.029
CIF Estimates
0.00
0.05
0.10
0.15
0
30
60
90
120
Time (Months)
Number at risk
1151 1133 1099 490 23
1855 1822 1764 1182 116
1060 1022 973 741 64
464 448 420 338 38
(B)
Outcome-Cardiovascular death
EA_score=0
EA_score=1
EA_score=2
EA_score>=3
p-value = 0.002
CIF Estimates
0.00
0.01
0.02
0.03
0.04
0
30
60
90
120
Time (Months)
Number at risk
1151 1133 1099 490 23
1855 1822 1764 1182 116
1060 1022 973 741 64
464 448 420 338 38
(C)
Outcome-Unexplained death
EA_score=0
EA_score=1
EA_score=2
EA_score>=3
p-value = 0.032
CIF Estimates
0.00
0.01
0.02
0.03
0
30
60
90
120
Time (Months)

Figure S3. Cumulative Incidence Function plots to compare the cumulative incidence of subjects with EA scores (0,1,2,3). P-values were calculated using Gray's test to elucidate whether significant differences exist in the different groups. (A) all-cause death. (B) CV death. (C) unexplained death.

(A)

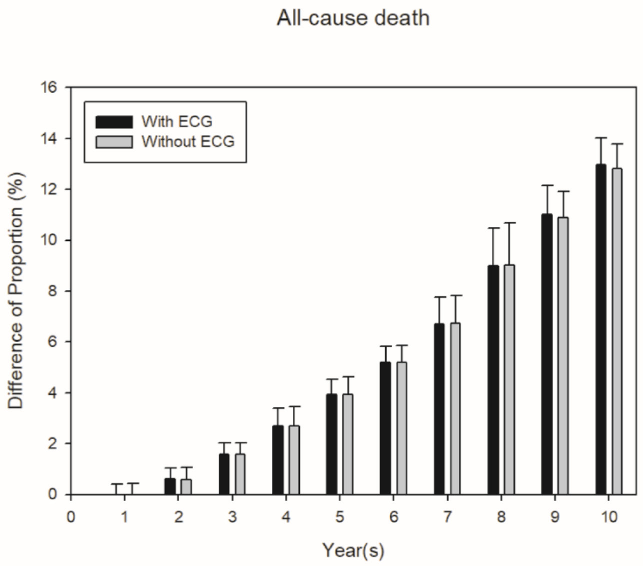

All-cause death
With ECG
Without ECG
Difference of Proportion (%)
0
2
4
6
8
10
12
14
16
0
1
2
3
4
5
6
7
8
9
10
Year(s)

(B)

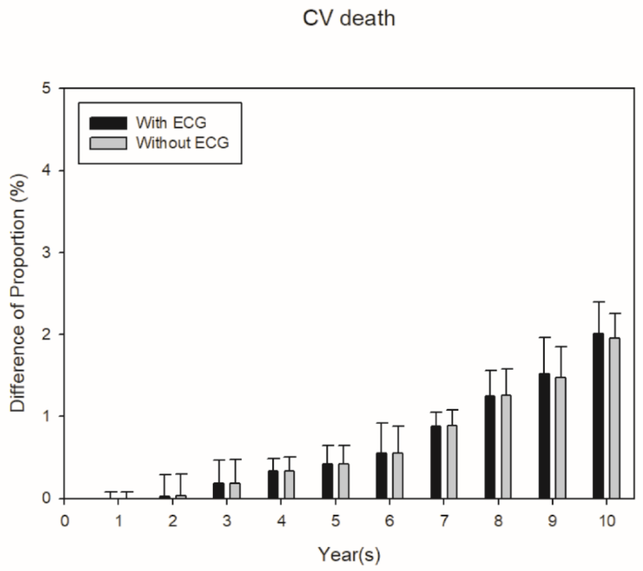

CV death
With ECG
Without ECG
Difference of Proportion (%)
0
1
2
3
4
5
0
1
2
3
4
5
6
7
8
9
10
Year(s)

(C)

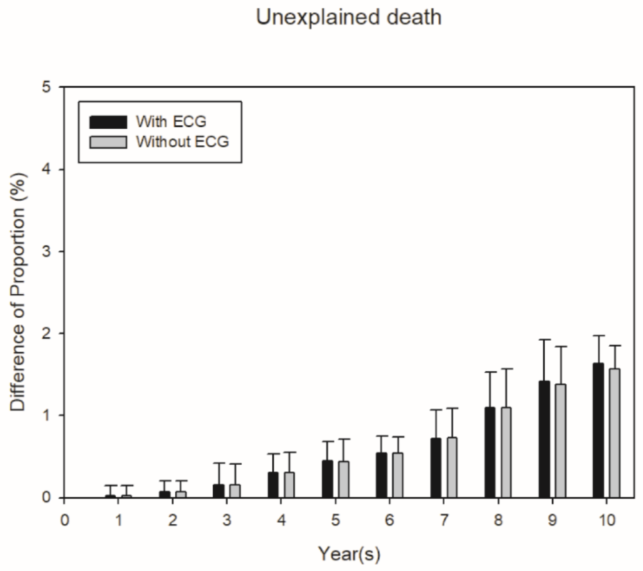

Unexplained death
With ECG
Without ECG
Difference of Proportion (%)
0
1
2
3
4
5
0
1
2
3
4
5
6
7
8
9
10
Year(s)

Figure S4. Calibration plots for the events (A) all-cause death, (B) cardiovascular death and (C) unexplained death, to show the difference between observed and predicted survival probability for proposed model (with EA score (0,1,2,3) and traditional variables) and traditional model (only traditional variables without EA score). Calibration for each of the models is conducted using 10-fold cross-validation (CV) and each bar shows an average of the probability difference over 10 models for each CV. Black bars: model with ECG + traditional variables. Grey bars: model with only traditional variables
